# Ensemble learning for the early prediction of neonatal jaundice with genetic features

**DOI:** 10.1186/s12911-021-01701-9

**Published:** 2021-12-01

**Authors:** Haowen Deng, Youyou Zhou, Lin Wang, Cheng Zhang

**Affiliations:** 1 School of Management, Fudan University, Shanghai, China; 2 Shanghai University of Finance and Economics, Shanghai, China; 3 Institutes of Biomedical Sciences, Fudan University, Shanghai, China

**Keywords:** Hyperbilirubinemia, Machine learning, Genetic variants, Transcutaneous bilirubin

## Abstract

**Background:**

Neonatal jaundice may cause severe neurological damage if poorly evaluated and diagnosed when high bilirubin occurs. The study explored how to effectively integrate high-dimensional genetic features into predicting neonatal jaundice.

**Methods:**

This study recruited 984 neonates from the Suzhou Municipal Central Hospital in China, and applied an ensemble learning approach to enhance the prediction of high-dimensional genetic features and clinical risk factors (CRF) for physiological neonatal jaundice of full-term newborns within 1-week after birth. Further, sigmoid recalibration was applied for validating the reliability of our methods.

**Results:**

The maximum accuracy of prediction reached 79.5% Area Under Curve (AUC) by CRF and could be marginally improved by 3.5% by including genetic variant (GV). Feature importance illustrated that 36 GVs contributed 55.5% in predicting neonatal jaundice in terms of gain from splits. Further analysis revealed that the main contribution of GV was to reduce the false-positive rate, i.e., to increase the specificity in the prediction.

**Conclusions:**

Our study shed light on the theoretical and practical value of GV in the prediction of neonatal jaundice.

**Supplementary Information:**

The online version contains supplementary material available at 10.1186/s12911-021-01701-9.

## Introduction

Neonatal jaundice is present in approximately 60% of term and 80% of preterm newborns [1]. Although most jaundice is benign, unexpected high bilirubin may occur and even cause permanent neural damage in newborns, i.e., “chronic bilirubin encephalopathy” or kernicterus. During the first week of life, an increase in bilirubin production and a decrease in bilirubin elimination cause total serum bilirubin (TsB) to rise rapidly [2, 3]. Therefore, jaundice, which may be preventable, is the leading cause of readmission during that period [4]. Pediatricians and scientists have been working on the prediction method of neonatal hyperbilirubinemia for decades. Most studies predicted neonatal jaundice through logistic regression [3, 5–9]. Other new methodologies included machine learning techniques to improve diagnosis in neonatal jaundice [10, 11].

Other studies also showed the association between functional variants and neonatal jaundice or bilirubin levels [12–18]. For instance, Uridine Diphosphate Glucuronosyl Transferase 1A1 (UGT1A1) has been identified as the key enzyme for bilirubin conjugation, while unconjugated bilirubin is the main cause of hyperbilirubinemia. Heme Oxygenase-1 (HMOX1) is another key enzyme in the bilirubin metabolism pathway for heme degradation [19]. Variants of *UGT1A1* and *HMOX1* were extensively studied, including (TA)n repeats in promoter and rs4148323 (G211A, Gly71Arg) in exon 1 in *UGT1A1*, and (GT)n repeats in promoter in *HMOX1*. However, few studies effectively utilized high-dimensional genetic features for neonatal jaundice prediction. One plausible reason could be the high discretion of GV that leads to large deviations in prediction. The challenges become more serious as genes are high-dimensional. As traditional methods require transferring multi-dimensional nominal variables into binary variables (i.e., one-hot encoding), they lose partial information to deal with a mass of GV and thus are inefficient. However, the association studies may estimate the prevalence in the general gene but lack the effectiveness to predict individual jaundice through integrating gene and clinical data.

**Fig. 1:**
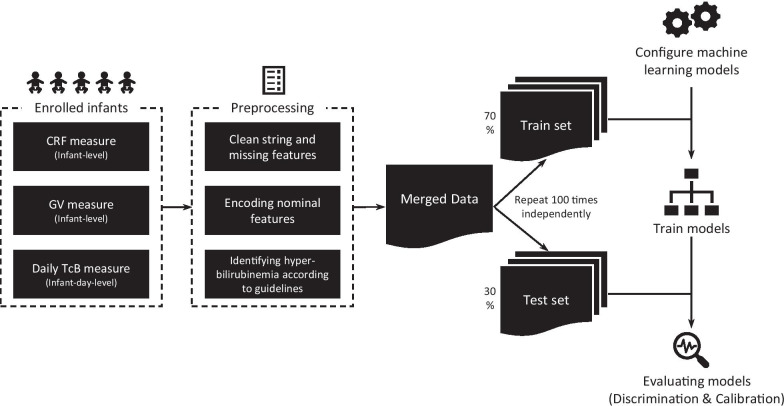
The methodological workflow of our study

This study applied an ensemble learning approach in machine learning to enhance the predictability of high-dimensional genetic features and CRF for physiological neonatal jaundice of full-term newborns within 1-week after birth. Using a data set from a municipal hospital in China, clinical predictors alone, genetic predictors alone, and clinical plus genetic predictors were tested separately by various machine learning (ML) techniques. We sought to create an ensemble learning approach to predict neonatal hyperbilirubinemia development so that pediatricians and parents may have more robust reference information before making decisions. The workflow of this study was summarized in Fig. 1.

## Method

### Study cohort

This study retrospectively enrolled 3743 infants born between February and October in 2008 at ≥ 37 weeks’ gestational age in Suzhou, China. Among them, 984 infants were randomly chosen from 3743 samples by matching gender, delivery mode and birth season for genotyping. Blood samples for genotyping were obtained from surplus filter papers, which were kept at 4 °C after routine newborn screening. Details of the genotyping procedure are in Additional file 1: Appendix 1. F-test showed there were no significant differences between the genotyped and un-genotyped samples in other major clinical characteristics, gestational age (F-value = 0.941, p = 0.238), and birth weight (F-value = 1.041, p = 0.455).

Eligible infants had no major abnormalities, except for neonatal jaundice without pathological causes, such as hemolytic disease of the newborn, glucose-6-phosphate dehydrogenase (G6PD) deficiency, and infection. Each neonate’s gender, birthday, delivery mode, gestational age at birth, birth weight, birth month, and feeding type were recorded. Transcutaneous bilirubin (TcB) was measured every morning on each neonate's forehead during birth hospitalization stay, resulting in a total of 4,048 records at the individual-day level (Table 1). Details of the measurement have been previously described [17]. According to Chinese guidelines in Practical Neonatology [20] and Practical Pediatrics [21], neonates were diagnosed as hyperbilirubinemia when their TcB exceeded 12.9 mg/dL (220.5 μmol/L) on day three or later days before they were discharged (namely CN220 in the study). Hyperbilirubinemic neonates would receive phototherapy. Bilirubin measurements within 24 h after phototherapy were excluded. Once the infants developed a high concentration of bilirubin before day three or the pathological cause of hyperbilirubinemia was diagnosed, such as hemolytic disease of the newborn, G6PD deficiency and infection, et al., infants would be transferred to the Neonatal Unit and excluded from our study. For internal missing measurements of TcB for a newborn, we imputed them with the average value of the previous and the next TcB levels.

**Table 1:** Descriptive summary of daily TcB levels (μmol/L)

Age (day)	Min	25%	Mean	Mode	75%	Max	Std	n	Ratio (%)
1	0	0	1.2	0	2.1	3.9	1.2	128	13
2	0	44.5	65.2	54.7	85.5	186.4	30.7	941	95.6
3	0	102.6	128.1	119.7	153.9	270.2	37.4	973	98.9
4	20.5	141.9	168.3	205.2	194.9	307.8	42.7	964	98
5	0	157.7	181.7	205.2	206.9	302.7	44.6	730	74.2
6	0	145.4	172.8	205.2	205.2	290.7	48.6	297	30.2
7	0	141.9	166.7	196.6	201.8	256.5	54.1	105	10.7

Ratio denotes the fraction of samples

DNA was isolated from surplus filter paper blood spots with ethanol. A set of 9 variants of *Uridine Diphosphate Glucuronosyl Transferase 1A1 (UGT1A1*), 4 variants of *Heme Oxygenase-1* (*HMOX1*), 6 variants of *Biliverdin Reductases A (BLVRA)* and 17 variants of *Solute Carrier Organic Anion Transporter family member 1B1* (*SLCO1B1*) was selected for genotyping. They were either functional SNPs or tagging SNPs in the genes of the enzymes in the bilirubin metabolism pathway; we integrated them as GV36 in the main analysis as additional predictors given CRF. Details of the genotyping method have been previously described [17].

### Predictors and outcome variables

Predictors included 6 CRF variants that were mostly mentioned in previous studies [10, 11], 4 *HMOX1* variants, 9 *UGT1A1* variants, 6 *BLVRA* variants, and 17 *SLCO1B1* variants. Descriptive statistics of CRF and major genetic variants are shown in Additional file 1: Appendix 2 Table A1 and Table A2, respectively.

**Table 2:** Thresholds to start phototherapy and the number of neonates exceeds the threshold (n +) according to different guidelines

Age (day)	CN220		NICE		P95		Sample size
	Thresholds	n+	Thresholds	n+	Thresholds	n+	
2	220	0	100	110	119.7	52	**943**
3	220	4	150	282	186.4	50	**979**
4	220	65	200	212	239.4	51	**969**
5	220	107	200	289	256.5	45	**747**
6	220	35	200	105	248.6	16	**305**
7	220	10	200	30	186.4	6	**105**

The best method is marked in bold with respect to each metric

The outcome variables are binary indicators that take on one if a newborn is hyperbilirubinemia. For generalizability purposes, this study also referred to other guidelines besides CN220, including NICE and P95, to evaluate the gene’s predictive power. NICE guidance was published by the UK’s National Institute for Health and Clinical Excellence in 2010. It recommended thresholds to start phototherapy according to hour-specific bilirubin level [22]. We took the first risk level of NICE as a comparable guideline threshold, denoted as NICE_R1. P95 refers to bilirubin levels at or greater than the 95th percentile of the population on the corresponding age. 95% percentile is commonly designated as high-risk zones. Such an idea was first suggested in 1999 [23]. It became popular after the American Academy of Pediatrics (AAP) applied the P95 risk zone in its updated guideline in 2004 [24]. Except for CN220, the other two guidelines’ bilirubin thresholds are age-specific. Daily bilirubin levels are descriptively summarized in Table 1. Table 2 summarizes the thresholds of bilirubin levels under different guidelines with the number of samples that exceed the thresholds.

### Ensemble learning

**Fig. 2:**
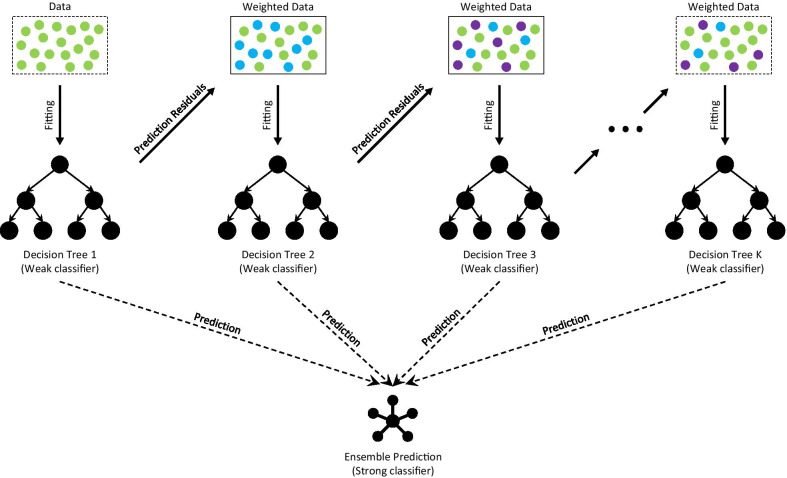
The architecture of Gradient Boosting Decision Tree

In machine learning, ensemble learning refers to the methods that use multiple learning algorithms to obtain better predictive performance than could be obtained from any of the single learning algorithms alone [25]. The ensemble learning framework was built on the gradient boosting decision tree (GBDT) that has a wide range of commercial and academic applications [26, 27]. To be specific, gradient boosting (GB) framework constructs additive regression models by sequentially fitting a weak classifier to current residuals [28, 29], as shown in Fig. 2. Thus, newly trained weak classifiers will correct the previous weak classifiers’ misjudgment, adaptively improving the prediction performance with high efficiency [30]. The final model aggregates the results from all weak classifiers to achieve a “strong” classifier as an ensemble. And GBDT is exactly the GB that utilizes decision trees as the weak classifiers, with a loss function to detect the residuals, such as mean squared error for regression or logarithmic loss for classification. By using 71 data sets originating from different domains and publicly available at UCI and KEEL repositories, GBDT exceeds or matches the prediction performance of other 10 popular algorithms for classification, including support vector machines, deep neural network, feedforward neural network, random forests, naïve Bayes, logistic regression and so on, and achieve the best accuracy ranking overall [31].

In the study, we implemented GBDT based on Lightgbm, a gradient boosting framework originally developed by Microsoft, which has shown its power in reducing the prediction bias in biology and computer science in recent years [32, 33]. To solve the high-dimensionality problem, we implemented lightgbm with L1 regularization [34], bagging [35] on samples (bootstrapping), and bagging on features.

To benchmark the model's prediction accuracy, we applied logistic regression (with L2 regularization), random forest, classification and regression tree (CART), and naïve Bayes method. All machine learning algorithms were implemented in Python, and the code is available in online resources.

### Evaluation

Following related frontier studies, this study used AUC on the test set as the metric of prediction. We took cross validation (CV) [36] with 30% samples as validation sets. As the incidence of neonatal hyperbilirubinemia is about 5% in practice, resulting in an unbalance problem that positive sample rates might be sensitive to sampling seed. Therefore, we controlled the positive sample ratio in each (train, validation) couple to be the same during sampling. The external validation was independently repeated 100 times for eliminating sampling bias in evaluating model performance. No hyperparameter tuning was applied based on the external cross-validation. For ensemble methods, i.e., lightgbm and random forests, internal bootstrapping (bagging) was applied for hyperparameter tuning and dealing with overfitting.

There is increasing attention to the calibration analysis to verify the reliability of risk prediction models to support medical decision-making [37]. A common definition of calibration is “having an event rate of *R*% among patients with a predicted risk of *R*%”. To verify the reliability of models, we calculated brier scores and plot calibration curves. Brier score is the estimated calibration index that builds on a flexible calibration analysis by computing the average squared difference between predicted risk and observed risk and transforming to obtain a value between 0 and 1 [38]. The lower the Brier score, the more reliable the prediction.

## Results

### Discrimination analysis

Across all neonatal jaundice guidelines, our ensemble learning method (lightgbm) achieved a high level of accuracy in terms of AUC based on clinical risk factors and genetic variants (CN220: see Table 3, other guidelines: see Additional file 1: Appendix 2 Table A3) superior to other non-ensemble methods. Performance metrics including accuracy, recall, and specificity were also evaluated in Additional file 1: Appendix 2 Table A4. Results indicated that lightgbm generally outperformed other machine learning algorithms in term of prediction. For the guideline implemented in our study, i.e., CN220, lightgbm classified the newborns with average AUC 0.792 (95% CI 0.757–0.828) based on only clinical risk factors. With the integration of 36 genetic variants (GV36), the accuracy retained a stronger performance level, i.e., AUC 0.82 (95% CI 0.785–0.857). To illustrate, GV36 contributed marginally AUC 0.028, about 3%, showing the effectiveness of lightgbm in utilizing high-dimensional genetic information into neonatal jaundice prediction. The marginal contribution of GV36 was consistent across guidelines and respectively achieved 0.036 for NICE_R1, 0.029 for P95.

**Table 3:** Discrimination results of predicting neonatal jaundice with CRF and GV under CN220 guideline

Variables	Method	AUC	F1-score	Precision
CRF	Lightgbm	**0.792** (0.757–0.828)	**0.213** (0.171–0.251)	0.136 (0.109–0.161)
	Cart	0.553 (0.509–0.592)	0.150 (0.074–0.211)	**0.137** (0.074–0.191)
	Logistic	0.785 (0.753–0.821)	0.210 (0.178–0.240)	0.122 (0.103–0.141)
	Naive Bayes	0.735 (0.673–0.782)	0.165 (0.129–0.188)	0.091 (0.069–0.104)
	rf	0.766 (0.711–0.806)	0.206 (0.177–0.245)	0.123 (0.106–0.147)
GV36	Lightgbm	**0.603** (0.546–0.662)	**0.149** (0.105–0.189)	0.105 (0.074–0.131)
	Cart	0.558 (0.522–0.598)	**0.149** (0.105–0.191)	**0.110** (0.079–0.139)
	Logistic	0.569 (0.519–0.614)	0.118 (0.093–0.141)	0.068 (0.053–0.081)
	Naive bays	0.562 (0.509–0.622)	0.112 (0.106–0.116)	0.059 (0.057–0.062)
	rf	0.587 (0.522–0.652)	0.148 (0.104–0.197)	0.103 (0.074–0.136)
CRF_GV36	Lightgbm	**0.820** (0.785–0.857)	**0.277** (0.218–0.333)	**0.204** (0.160–0.247)
	Cart	0.569 (0.517–0.621)	0.184 (0.103–0.269)	0.175 (0.095–0.250)
	Logistic	0.781 (0.730–0.816)	0.218 (0.185–0.251)	0.129 (0.110–0.150)
	Naive Bayes	0.642 (0.563–0.707)	0.114 (0.105–0.124)	0.061 (0.056–0.067)
	rf	0.792 (0.753–0.833)	0.228 (0.193–0.259)	0.139 (0.118–0.158)

The best performance by algorithms with CRF, GV36 and CRF_GV36 variables are marked in bold

95% CI is shown in parentheses

**Table 4:** Calibration results of predicting neonatal jaundice with CRF and GV. 95%

Recali-brated	Guideline	Variables	AUC	Brier	Event rate	Average risk
No	CN220	CRF	0.792 (0.757–0.828)	0.054 (0.05–0.058)	0.055	0.047
		CRF_GV36	0.82 (0.785–0.857)	0.053 (0.05–0.057)	0.055	0.038
	NICE_R1	CRF	0.72 (0.695–0.744)	0.172 (0.164–0.179)	0.254	0.250
		CRF_GV36	0.756 (0.736–0.78)	0.165 (0.155–0.175)	0.254	0.244
	P95	CRF	0.68 (0.623–0.737)	0.053 (0.05–0.056)	0.054	0.048
		CRF_GV36	0.709 (0.657–0.773)	0.054 (0.049–0.06)	0.054	0.043
Yes	CN220	CRF	0.795 (0.761–0.83)	0.051 (0.049–0.052)	0.055	0.055
		CRF_GV36	0.83 (0.802–0.862)	0.049 (0.048–0.051)	0.055	0.055
	NICE_R1	CRF	0.724 (0.702–0.752)	0.168 (0.163–0.173)	0.254	0.254
		CRF_GV36	0.762 (0.739–0.787)	0.158 (0.152–0.164)	0.254	0.255
	P95	CRF	0.683 (0.622–0.733)	0.05 (0.049–0.052)	0.054	0.055
		CRF_GV36	0.717 (0.669–0.772)	0.049 (0.047–0.05)	0.054	0.055

95% CI is shown in parentheses

In addition to the strong performance of lightgbm, another ensemble learning method, random forest (RF), performed comparably well. Notably, RF even surpassed lightgbm in NICE_R1 and P95 if only predicting with GV36. Although RF didn’t achieve as well as lightgbm after additionally including genetic information, it also indicated that the marginal contribution of GV was consistent across guidelines, i.e., 0.026 for CN200, 0.036 for NICE_R1, and 0.029 for P95, which further validated the effectiveness of ensemble learning in integrating genetic variants into predicting neonatal jaundice.

While both ensemble tree algorithms (Lightgbm and RF) achieved high accuracy and effectively enhanced the prediction by integrating clinical risk factors and genetic information, a single tree (CART) failed to precisely predict neonatal jaundice. For example, CART achieves AUC 0.569 (95% CI 0.517–0.621) in CN220 guideline with CRF, far from that of lightgbm, i.e. 0.82 (95% CI 0.785–0.857). It indicated that the ensemble of weak classifiers could achieve outstanding performance in predicting neonatal jaundice.

Although traditional methods, logistics, and naïve Bayes achieved comparable accuracy with clinical risk factors, they could not benefit from genetic information and might even worsen. For instance, under CN220, logistic regression achieved 0.785 (95% CI 0.753–0.821) AUC, which decreased to 0.781 (95% CI 0.73–0.816) AUC after additionally including GV36 as explaining variables. We have implemented L2-regularization into the logistic regression as a common method to deal with overfitting and high-dimensionality.

**Fig. 3:**
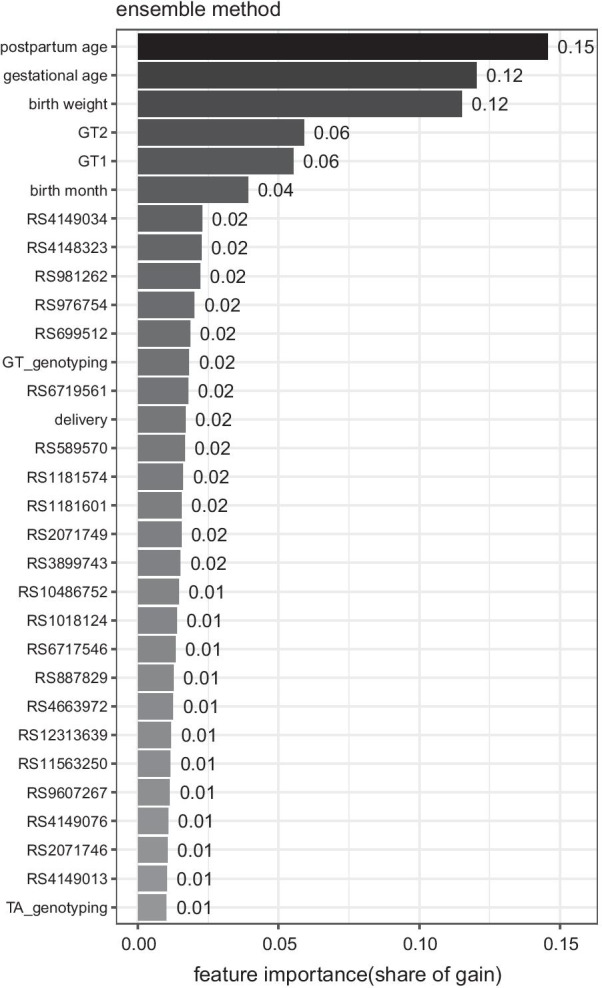
Relative feature importance from ensemble nethod in predicting neonatal jaundice under CN220 guideline

To gain insight into how the prediction system utilizes clinical risk factors and genetic information, we identified key clinical features and genetic variants driving the ensemble learning. Figure 3 showed the feature importance of our ensemble method (lightgbm) measured by *gain* from splits under the representative guideline: CN220. The overall feature importance of CRF covered 44.5%, while GV contributed 55.5% in predicting neonatal jaundice in terms of *gain*.

### Calibration analysis

**Fig. 4:**
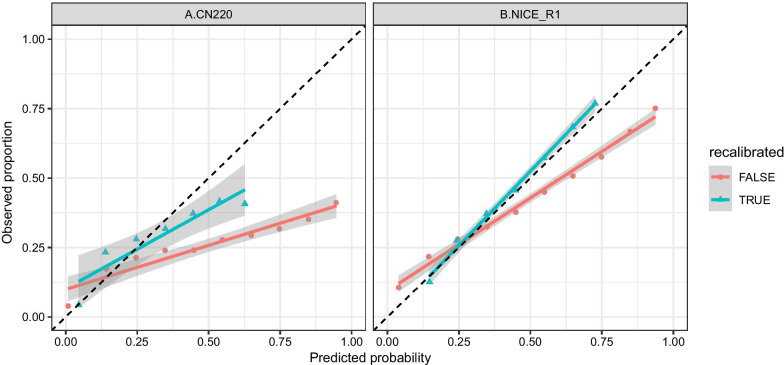
Calibration curves on external validation sets

Following previous studies [37, 39, 40], we investigated our method's calibration performance (lightgbm) based on calibration curves and brier score. Calibration curves (Fig. 4) showed the observed proportion of events associated with our model’s predicted risk [41], under CN220 and NICE_R1 guidelines. The red lines referred to the linearly fitted line of original calibration curves of lightgbm with 95% CI. Since the red lines deviated from the diagonal significantly, the model suffered from overfitting. Specifically, our method before recalibration tended to overestimate high risks and underestimate low risk for both guidelines.

To improve the reliability of our method, we implemented the sigmoid recalibration [42]. In particular, an additional sigmoid function was trained to map the Lightgbm outputs into recalibrated predictions based on 10-folder internal cross-validation on train sets. Recalibrated curves (green lines in Fig. 4) were significantly amended towards the diagonal lines, illustrating our method's moderate calibration level in predicting neonatal jaundice.

Further, brier scores gave quantitative measurements of calibration performance (Table 4). It indicated that sigmoid recalibration improved the calibration performance in terms of brier scores and enhanced the discrimination performance in terms of AUC. For instance, under CN220 guideline, lightgbm obtained an average brier score 0.053 (95% CI 0.05–0.057) and an average AUC 0.82 (95% CI 0.785–0.857) with CRF and genetic variants. After recalibration, the corresponding brier score was improved to 0.049 (95% CI 0.048–0.051), while the recalibrated AUC was 0.83 (95% CI 0.802–0.862). Meanwhile, GV’s additional contribution was enhanced to 0.035 for CN220, 0.038 for NICE_R1, 0.034 for P95. After recalibration, the average event rates were matched with the average prediction risks, which were not before recalibration. Therefore, recalibration could further enhance our method’s reliability in individual-level implementation.

### Robustness checks

We experimented with the prediction by using a different combination of GV, as shown in Table 5. We chose 4 GV out of 36 according to the popularity and feature importance, denoted as GV4. In addition to (TA)n repeat, rs4148323 (G211A, Gly71Arg) and (GT)n repeat, rs887829 (c-364t) in *UGT1A1* were shown to be associated with adults’ bilirubin level [43]. Additionally, we chose 7 GVs that were tagging SNPs located within 5 kb upstream and 2 kb downstream of each gene, selected from the HapMap Han Chinese population based on r^2^ > 0.8 and a minor allele frequency of > 0.1. The 7 GVs were integrated into GV4 to obtain GV11. In this way, we can compare the change of prediction accuracy with 4, 11, and 36 GV.

**Table 5:** Prediction performance of recalibrated lightgbm under CN220 guideline with different combinations of GV

Variables	AUC	F1-score	Precision	Specific GV
CRF	0.795 (0.761–830)	0.217 (0.171–0.261)	0.143 (0.113–0.171)	None
CRF + GV4	0.807 (0.779–841)	0.242 (0.195–0.286)	0.165 (0.132–0.194)	*HMOX1* (1) rs2071749 *UGT1A1* (3): rs4148323, rs6717546, rs6719561
CRF + GV11	0.813 (0.781–0.847)	0.251 (0.198–0.298)	0.176 (0.141–0.207)	*HMOX1* (3) (GT)n, rs9607267, rs2071749 *UGT1A1* (8) rs887829, (TA)n, rs4148323, rs1018124, rs6717546, rs11563250, rs6719561, rs4663972
CRF + GV36	0.830 (0.802–0.826)	0.285 (0.229–0.333)	0.217 (0.173–0.252)	*HMOX1* (4) rs2071746, (GT)n, rs9607267, rs2071749 *UGT1A1* (9) rs4399719, rs887829, (TA)n, rs4148323, rs1018124, rs6717546, rs11563250, rs6719561, rs4663972 *BLVRA* (6) rs1181601, rs1181574, rs10486752, rs699512, rs17246016, rs589570 *SLCO1B1* (17) rs4149013, rs10743408, rs3899743, rs981262, rs7138177, rs4149026, rs976754, rs4149034, rs12313639, rs2306283, rs4149044, rs4149056, rs4149057, rs4363657, rs4149076, rs12578392, rs4149085

95% CI is shown in parentheses

**Fig. 5:**
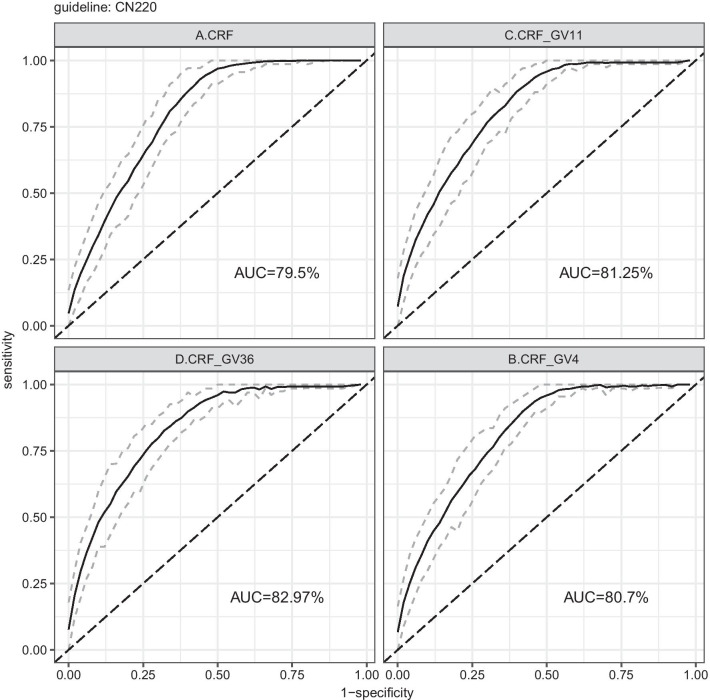
ROC curve of neonatal jaundice prediction with CRF and GV by ensemble learning

Results of recalibrated lightgbm under CN220 guideline with different combinations of GV (Table 5 and Fig. 5) showed that the additional improvement by using 4, 11, 36 GV were respectively 0.011, 0.016, and 0.029 AUC with the ensemble method. It indicated that a small subset of GV (GV4) could achieve about 1/3 additional predictive power of GV36, and the marginal contribution of GV11 covers about a half of that of GV36, which facilitated the clinical application of GV by lowering requirements of gene quantity for saving costs. The 0.035 additional enhanced prediction power by GV36 also suggested a mass of reserve force of gene for predicting neonatal jaundice and waiting for being discovered in the future.

### Extended analysis

**Fig. 6:**
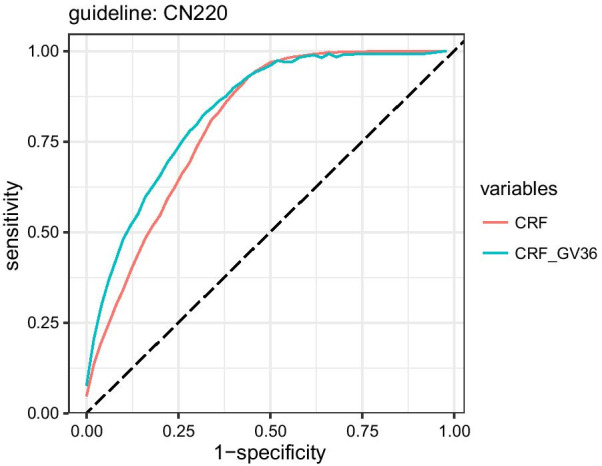
Comparison of ROC curve of neonatal jaundice prediction after introducing genetic variants (GV36)

To gain a deeper understanding of gene variables' contribution to predicting neonatal jaundice, we mapped the ROC curve of the model with GV and CRF as independent variables, as shown in Fig. 6. It showed that when using CRF alone, true positive rate (TPR, i.e., sensitivity) reached 1 when the False positive rate (FPR, i.e., 1-Specificity) is about 0.5, indicating that the CRF is more conducive to improving the TPR; when incorporating GV to CRF, the ROC curve is further extended to the left, indicating that the main contribution of GV is to reduce the FPR and increase the specificity. Therefore, it is plausible to argue that GV's clinical contribution on increasing the prediction accuracy of physiological neonatal jaundice is mainly to avoid misdiagnosis due to false positives.

## Discussions

The contribution of this study is the incorporation of high-dimensional GV for predicting neonatal jaundice effectively. We showed that integrating GV with CRF can further improve the discrimination performance by 3.5% (CN220) AUC and 3.8% (NICE) AUC than using CRF alone. Further, we deduced GV's relative importance and explanatory power, which provides quantitative support for further experimental validation of gene variants' mechanism in neonatal jaundice. Our study's potential clinical application is to estimate the probability of neonatal jaundice within one week after birth.

Our results show that our method can effectively improve the upper limit of CRF’s prediction by integrating it with gene features, thus opening up a new way for the clinical diagnosis of neonatal jaundice with GV. The study further reveals that although more gene information can better help clinical diagnosis, the GV contributes differently to the prediction. In this way, only a small amount of genetic information is needed in practice to predict neonatal jaundice effectively.

Different from the early bilirubin level, the genetic features have been determined since the embryo period. Consequently, the study obtains a clinical application advantage compared with existing literature that uses early bilirubin level into prediction: the model predicts the risk of neonatal jaundice for discharged newborns before any bilirubin level measurement coming out. Furthermore, its prediction power does not rely on repeated bilirubin level measurement, making the prediction more convenient and efficient than previous ones.

For newborns within one week, bilirubin measurements are repeated several times. Lightgbm and random forests are based on decision tree algorithm, which does not assume a functional relationship between the outcome and features. Thus, our method is flexible towards the assumption of Independent and Identically Distributed (IID) in predicting neonatal jaundice.

The study is not free from limitations. First, all bilirubin levels are measured within one week after birth. Thus, the scope of the clinical application might be limited. Second, although TcB is a good index for a non-invasive auxiliary diagnostic system and TcB correlates well with TsB, the correlation might not be stable at high-level bilirubin concentrations [44], the findings in the study may not apply to TsB prediction directly. Future research can consider TsB as a prediction target by using GV and CRF features together.

## Conclusion

In summary, this paper applied an ensemble learning method (lightgbm) to integrating 36 GVs into predicting neonatal jaundice, measured by TcB. Results demonstrated that our method effectively solved the technical difficulties on GV’s high dimensionality. Quantitatively, GV contributes an additional 3.5% AUC based on prediction with CRF after sigmoid recalibration. Although the best predictors were CRF, GV was exactly complementary no matter which guideline to take. The study sheds light on the clinical importance and effective approach of how to facilitate predicting neonatal jaundice with high-dimensional GV. With the popularization of medical big data and the improvement of gene sequencing technology, the risk assessment and research of neonatal diseases with the gene will be fully developed.

## Supplementary Information

**Additional file 1.** The file containing **Appendix 1:** Genotyping Method, and **Appendix 2:** supplementary tables including Table A1 to A4.

## Data Availability

The datasets used and/or analyzed during the current study are available from the corresponding author on reasonable request.

## Abbreviations

CRF: Clinical risk factors
GV: Genetic variants
GBDT: Gradient boosting decision tree
RF: Random forests
AUC: Area Under the Curve
NICE: The guidance published by the UK’s National Institute for Health and Clinical Excellence
P95: The guidance with the threshold 95% percentile
CN220: The Chinese guidance with the threshold 220.5 μmol/L
TcB: Transcutaneous bilirubin
