# Supplementary material for: Ensemble learning for the early prediction of neonatal jaundice with genetic features
Source: BMC Med Inform Decis Mak. 2021 Dec 1;21:338. doi: 10.1186/s12911-021-01701-9 (PMC8638201; doi:10.1186/s12911-021-01701-9)
Supplement: Supplementary file 1 — Additional file 1. The file containing Appendix 1: Genotyping Method, and Appendix 2: supplementary tables including Table A1 to A4. [file 12911_2021_1701_MOESM1_ESM.docx]

Appendix 1: Genotyping Method

The study was approved by both Suzhou Municipal Hospital Reproductive Medicine Ethics Committee and the Ethics Committee of Institutes of Biomedical Sciences. Since the data were analyzed anonymously, the filter paper was obtained from a standard screening procedure, and the TcB measurement was a completely noninvasive routine clinical assessment, both committees approved a waiver of written consent. Each filter paper was placed in an Eppendorf tube, fully soaked in 16PBS (pH =8.0), and incubated in boiled water for 5 minutes. After a quick spin, the supernatant was transferred to a new tube, washed with absolute ethanol, centrifuged at 13,000 rpm for 10 minutes, and the supernatant was discarded. After a second wash with 70% ethanol, the precipitate was air dried. After resuspension in distilled H2O, the DNA extract of 11 genetic variants was kept at 220uC until further use.

Appendix 2: Supplementary tables

Table A1. Descriptive Statistics of CRF

| characteristic | value | freq | % |
| --- | --- | --- | --- |
| birth month |  |  |  |
|  | 2 | 18 | 1.8 |
|  | 3 | 99 | 10.1 |
|  | 4 | 75 | 7.6 |
|  | 5 | 125 | 12.7 |
|  | 6 | 88 | 8.9 |
|  | 7 | 37 | 3.8 |
|  | 8 | 155 | 15.8 |
|  | 9 | 194 | 19.7 |
|  | 10 | 193 | 19.6 |
| gender |  |  |  |
|  | male | 532 | 54.1 |
|  | female | 452 | 45.9 |
| delivery |  |  |  |
|  | Vaginal | 516 | 52.4 |
|  | Cesarean | 417 | 42.4 |
|  | Forceps | 51 | 5.2 |
| feeding |  |  |  |
|  | breast | 975 | 99.1 |
|  | mixed | 7 | 0.7 |
|  | formula milk | 2 | 0.2 |
| birth weight (g) |  |  |  |
|  | 2500 < 2975 | 123 | 12.5 |
|  | 2975 < 3450 | 478 | 48.6 |
|  | 3450 < 3925 | 282 | 28.7 |
|  | 3925 < 4400 | 82 | 8.3 |
|  | 4400 < 4875 | 13 | 1.3 |
|  | 4875 < 5350 | 6 | 0.6 |
| gestational age (days) |  |  |  |
|  | 259 < 266.2 | 101 | 10.3 |
|  | 266.2 < 273.3 | 242 | 24.6 |
|  | 273.3 < 280.5 | 292 | 29.7 |
|  | 280.5 < 287.7 | 218 | 22.2 |
|  | 287.7 < 294.8 | 113 | 11.5 |
|  | 294.8 < 302 | 18 | 1.8 |

Table A2. Descriptive Statistics of GV

| characteristic | value | freq | % |
| --- | --- | --- | --- |
| *HMOX1*-(GT)n |  |  |  |
|  | group1 (SS-SM)* | 595 | 60.5 |
|  | group2 (SL-MM-ML-LL) | 376 | 38.2 |
|  | Missing | 13 | 1.3 |
| *HMOX1*-rs9607267 |  |  |  |
|  | homozygous mutation  heterozygous mutation | 202  484 | 20.5  49.2 |
|  | wild type | 278 | 28.3 |
|  | Missing | 20 | 2.0 |
| *HMOX1*-rs2071749 |  |  |  |
|  | homozygous mutation  heterozygous mutation | 59  392 | 6.0  39.8 |
|  | wild type | 484 | 49.2 |
|  | Missing | 49 | 5.0 |
| *UGT1A1*-rs887829 |  |  |  |
|  | homozygous mutation  heterozygous mutation | 11  189 | 1.1  19.2 |
|  | wild type | 765 | 77.7 |
|  | Missing | 19 | 1.9 |
| *UGT1A1*-(TA)n |  |  |  |
|  | homozygous mutation  heterozygous mutation | 14  181 | 1.4  18.4 |
|  | wild type | 780 | 79.3 |
|  | Missing | 9 | 0.9 |
| *UGT1A1*-rs4148323 |  |  |  |
|  | homozygous mutation  heterozygous mutation | 28  307 | 2.8  31.2 |
|  | wild type | 614 | 62.4 |
|  | Missing | 35 | 3.6 |
| *UGT1A1*-rs1018124 |  |  |  |
|  | homozygous mutation  heterozygous mutation | 27  254 | 2.7  25.8 |
|  | wild type | 506 | 51.4 |
|  | Missing | 197 | 20.0 |
| *UGT1A1*-rs6717546 |  |  |  |
|  | homozygous mutation  heterozygous mutation | 64  399 | 6.5  40.5 |
|  | wild type | 461 | 46.8 |
|  | Missing | 60 | 6.1 |
| *UGT1A1*-rs11563250 |  |  |  |
|  | homozygous mutation  heterozygous mutation | 26  248 | 2.6  25.2 |
|  | wild type | 683 | 69.4 |
|  | Missing | 27 | 2.7 |
| *UGT1A1*-rs6719561 |  |  |  |
|  | homozygous mutation  heterozygous mutation | 126  417 | 12.8  42.4 |
|  | wild type | 390 | 39.6 |
|  | Missing | 51 | 5.2 |
| *UGT1A1*-rs4663972 |  |  |  |
|  | homozygous mutation  heterozygous mutation | 15  238 | 1.5  24.2 |
|  | wild type | 622 | 63.2 |
|  | Missing | 109 | 11.1 |

*Small allele (S) <27 GT repeats; Middle alleles (M): 27-32 GT repeats; Long alleles (L)≥33 GT repeats.

Table A3. Discrimination results of predicting neonatal jaundice with CRF and GV in terms of AUC, while 95% CI is shown in parentheses.

| guideline | variables | method | auc | 95% CI |
| --- | --- | --- | --- | --- |
| CN220 | CRF | lightgbm | 0.792 | (0.757-0.828) |
|  |  | cart | 0.553 | (0.509-0.592) |
|  |  | logistic | 0.785 | (0.753-0.821) |
|  |  | naive Bayes | 0.735 | (0.673-0.782) |
|  |  | rf | 0.766 | (0.711-0.806) |
|  | GV36 | lightgbm | 0.603 | (0.546-0.662) |
|  |  | cart | 0.558 | (0.522-0.598) |
|  |  | logistic | 0.569 | (0.519-0.614) |
|  |  | naive Bayes | 0.562 | (0.509-0.622) |
|  |  | rf | 0.587 | (0.522-0.652) |
|  | CRF_GV36 | lightgbm | 0.82 | (0.785-0.857) |
|  |  | cart | 0.569 | (0.517-0.621) |
|  |  | logistic | 0.781 | (0.73-0.816) |
|  |  | naive Bayes | 0.642 | (0.563-0.707) |
|  |  | rf | 0.792 | (0.753-0.833) |
| NICE_R1 | CRF | lightgbm | 0.72 | (0.695-0.744) |
|  |  | cart | 0.575 | (0.545-0.603) |
|  |  | logistic | 0.661 | (0.632-0.684) |
|  |  | naive Bayes | 0.613 | (0.587-0.652) |
|  |  | rf | 0.695 | (0.673-0.718) |
|  | GV36 | lightgbm | 0.677 | (0.648-0.702) |
|  |  | cart | 0.666 | (0.63-0.693) |
|  |  | logistic | 0.569 | (0.542-0.593) |
|  |  | naive Bayes | 0.545 | (0.516-0.568) |
|  |  | rf | 0.689 | (0.663-0.713) |
|  | CRF_GV36 | lightgbm | 0.756 | (0.736-0.78) |
|  |  | cart | 0.601 | (0.556-0.637) |
|  |  | logistic | 0.655 | (0.624-0.679) |
|  |  | naive Bayes | 0.565 | (0.531-0.59) |
|  |  | rf | 0.749 | (0.722-0.767) |
| P95 | CRF | lightgbm | 0.68 | (0.623-0.737) |
|  |  | cart | 0.567 | (0.527-0.617) |
|  |  | logistic | 0.578 | (0.48-0.635) |
|  |  | naive Bayes | 0.57 | (0.518-0.621) |
|  |  | rf | 0.675 | (0.621-0.733) |
|  | GV36 | lightgbm | 0.67 | (0.619-0.727) |
|  |  | cart | 0.646 | (0.601-0.695) |
|  |  | logistic | 0.573 | (0.522-0.627) |
|  |  | naive Bayes | 0.518 | (0.463-0.564) |
|  |  | rf | 0.696 | (0.637-0.753) |
|  | CRF_GV36 | lightgbm | 0.709 | (0.657-0.773) |
|  |  | cart | 0.594 | (0.553-0.642) |
|  |  | logistic | 0.584 | (0.526-0.635) |
|  |  | naive Bayes | 0.539 | (0.482-0.597) |
|  |  | rf | 0.705 | (0.658-0.765) |

Table A4. Comprehensive evaluation of 10 most popular machine learning methods in predicting neonatal jaundice, with all features (CRF, GV36) as explaining variables, under CN220 guideline.

| Method | AUC | | Accuracy | | Precision | | F1 score | | Specificity | | Recall | |
| --- | --- | --- | --- | --- | --- | --- | --- | --- | --- | --- | --- | --- |
| lightgbm | **0.820** | (0.785-0.857) | 0.875 | (0.860-0.891) | **0.204** | (0.160-0.247) | **0.277** | (0.218-0.333) | 0.901 | (0.885-0.918) | 0.433 | (0.328-0.537) |
| ab | 0.794 | (0.759-0.823) | 0.055 | (0.055-0.056) | 0.055 | (0.055-0.055) | 0.104 | (0.104-0.105) | 0.000 | (0.000-0.001) | **1.000** | (1.000-1.000) |
| rf | 0.793 | (0.752-0.826) | 0.762 | (0.742-0.786) | 0.138 | (0.118-0.158) | 0.227 | (0.193-0.255) | 0.769 | (0.748-0.794) | 0.633 | (0.530-0.720) |
| logistic | 0.781 | (0.732-0.817) | 0.721 | (0.683-0.757) | 0.129 | (0.110-0.150) | 0.218 | (0.185-0.251) | 0.722 | (0.677-0.760) | 0.705 | (0.597-0.814) |
| elm | 0.662 | (0.554-0.737) | 0.055 | (0.055-0.055) | 0.055 | (0.055-0.055) | 0.104 | (0.104-0.104) | 0.000 | (0.000-0.000) | **1.000** | (1.000-1.000) |
| naive Bayes | 0.642 | (0.563-0.707) | 0.285 | (0.231-0.353) | 0.061 | (0.056-0.067) | 0.114 | (0.105-0.124) | 0.252 | (0.193-0.330) | 0.839 | (0.724-0.940) |
| nn | 0.593 | (0.489-0.699) | 0.860 | (0.058-0.945) | 0.017 | (0.000-0.113) | 0.024 | (0.000-0.161) | 0.903 | (0.003-1.000) | 0.117 | (0.000-1.000) |
| knn | 0.584 | (0.528-0.633) | 0.766 | (0.739-0.787) | 0.094 | (0.070-0.119) | 0.151 | (0.110-0.190) | 0.789 | (0.760-0.814) | 0.377 | (0.261-0.478) |
| cart | 0.569 | (0.509-0.614) | **0.904** | (0.890-0.917) | 0.172 | (0.072-0.239) | 0.181 | (0.074-0.261) | **0.945** | (0.931-0.959) | 0.193 | (0.075-0.284) |
| svc | 0.531 | (0.479-0.583) | 0.320 | (0.098-0.698) | 0.057 | (0.048-0.069) | 0.105 | (0.088-0.119) | 0.297 | (0.048-0.722) | 0.722 | (0.284-0.970) |

Abbreviations of methods - ab: AdaBoost, rf: random forest, elm: extreme learning machine, nn: neural network (or called multi-layer perceptron classifier), knn: k-nearest neighborhood votes, cart: classification and regression tree, svc: support vector machine classifier. All algorithms were coded in python based on packages: lightgbm, sklearn and numpy.
